# Cardiac Metastasis of Malignant Melanoma Detected by ^18^F-FDG PET/CT

**DOI:** 10.3390/diagnostics13132234

**Published:** 2023-06-30

**Authors:** Hongzhe Zhang, Yaping Luo

**Affiliations:** 1 Department of Nuclear Medicine, Chinese Academy of Medical Sciences and Peking Union Medical College Hospital, Beijing 100730, China; 2 Beijing Key Laboratory of Molecular Targeted Diagnosis and Therapy in Nuclear Medicine, Beijing 100730, China

**Keywords:** cardiac metastasis, malignant melanoma, ^18^F-FDG, PET/CT

## Abstract

A 63-year-old man with a history of right plantar malignant melanoma (T3bN0M0, IIb) developed multiple metastases in bilateral lungs 19 months after surgery. Subsequent ^18^F-FDG PET/CT revealed multiple pulmonary metastases with intense FDG uptake and detected a hypermetabolic lesion in the lateral wall of the left ventricle, which was considered to be a cardiac metastasis of malignant melanoma. This lesion was later confirmed in the dynamic myocardial perfusion MR. This case demonstrates the effectiveness of ^18^F-FDG PET/CT in detecting occult cardiac metastases of malignant melanoma.

Cardiac metastases are rare, occurring in 4.71% of adult patients with solid cancers, and lung cancer accounted for most primary tumors. In terms of locations, the pericardium was the most frequent site of cardiac metastases, followed by myocardial metastases [1]. Cardiac involvement of malignant melanoma was reported to occur in less than 2% of patients. As most patients are asymptomatic, cardiac metastasis from melanoma is usually found in autopsy [2,3] or detected opportunistically [4,5], and it was associated with poor prognosis compared to those without cardiac metastases [6,7]. The sensitivity of routine cardiac examinations for cardiac metastasis is limited [8]. ^18^F-FDG PET/CT is a useful tool to assess distant metastases of melanoma, including detecting occult cardiac metastases [9]. However, myocardium usually presents variable physiological ^18^F-FDG uptake [10], and cardiac lesions may often be overlooked. Therefore, reducing the physiological ^18^F-FDG uptake in the myocardium is necessary for this situation. Several methods for inhibiting physiological myocardial ^18^F-FDG uptake have been proposed, including long fasting (longer than 18 h) [11], a low-carbohydrate diet [12] or prolonged high-fat, high-protein, and very-low-carbohydrate diet [13]. These procedures may lead to an increase in circulating free fatty acid (FFA) levels as well as a decrease in blood insulin. However, there is no definite conclusion as to which procedure is optimal, and the choice needs to be made based on clinical practice. Nuclear medicine physicians should keep this in mind when reading images with abnormal cardiac uptake and be aware of the procedures to suppress the background uptake of the myocardium.

**Figure 1:**
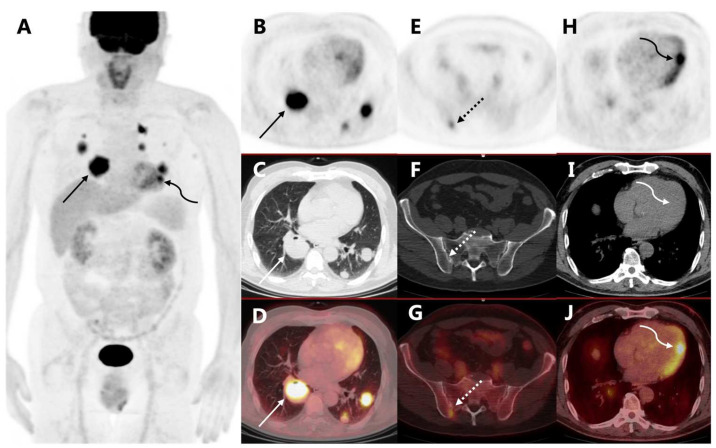
A 61-year-old man was recently diagnosed with cutaneous melanoma in the right planta (Breslow thickness 2 mm). The patient had no family history of malignancy. Preoperative ^18^F-FDG PET/CT did not show any nodal or distant metastasis. He then underwent enlarged excision of the tumor and sentinel lymph-node biopsy, and his tumor was staged T3bN0M0 (IIb). He did not receive adjuvant treatment and was closely followed postoperatively. Nineteen months after surgery, he presented with blood-stained sputum and CT revealed multiple masses and nodules in bilateral lungs, which were confirmed to be pulmonary metastases from malignant melanoma after biopsy. The immunohistochemical results were AE1/AE3 (−), HMB45 (+), Melan-A (+), Ki-67 (index 50%), TTF-1 (−), S-100 (+). The follow-up ^18^F-FDG PET/CT ((**A**) PET MIP, (**B**) axial PET, (**C**) coregistered CT, (**D**) fusion image) showed multiple hypermetabolic masses in bilateral lungs (arrows, SUVmax 10.6), consistent with pulmonary metastases. Bone metastasis in the right ilium was also detected ((**E**)–(**G**), dotted arrows, SUVmax 3.1). His left ventricular myocardium exhibited diffuse uptake, which was physiological (Figure 1). Notably, a nodular hypermetabolic lesion was found in the lateral wall of the left ventricle ((**H**)–(**J**), curved arrows, SUVmax 7.1), which was abnormal, possibly a cardiac metastasis from melanoma.

**Figure 2:**
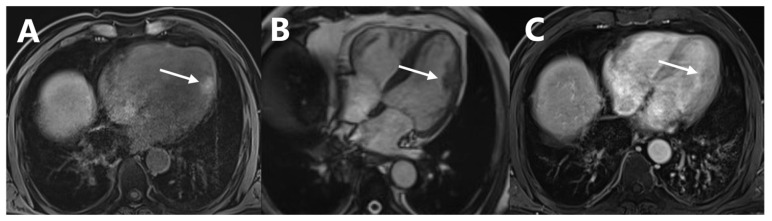
Dynamic myocardial perfusion MRI ((**A**) T1WI, (**B**) T2WI, (**C**) delayed scan of enhanced T1WI) also detected the lesion in the left ventricular lateral wall with a short T1 signal and a long T2 signal intensity with delayed enhancement (Figure 2, arrows). Subsequent gene testing revealed an insertion mutation in exon 11 of the KIT gene and no BRAF mutations were detected. Considering the advanced stage of malignant melanoma, he was treated with imatinib and received radiotherapy of the largest mass in the right lung. However, the treatment of imatinib was discontinued 6 weeks later because of a drug reaction with eosinophilia and systemic symptoms (DRESS). The patient experienced rapid progression of the tumor 4 months thereafter and died of the disease.

## Data Availability

No new data were created or analyzed in this study. Data sharing is not applicable to this article.
